# High-Performance Paper-Based Capacitive Flexible Pressure Sensor and Its Application in Human-Related Measurement

**DOI:** 10.1186/s11671-019-3014-y

**Published:** 2019-05-29

**Authors:** Weizhi Li, Li Xiong, Yueming Pu, Yong Quan, Shibin Li

**Affiliations:** State Key Laboratory of Electronic Thin Films and Integrated Devices, School of Optoelectronic Science and Engineering, University of Electronic Science and Technology of China, Chengdu, 610054 China

**Keywords:** Capacitive flexible pressure sensor, Silver nanowires, PDMS, Wearable devices

## Abstract

Flexible pressure sensors (FPS) have shown wide applications in artificial robotics, wearable devices, electronic skins, and biomedical systems; however, complicated procedures like micromachining and micromolding are often involved to achieve high performance of the sensor. In this work, a novel capacitive FPS was prepared by using silver nanowire (AgNW)-paper substrates as electrodes and polydimethylsiloxane (PDMS) as dielectrics, and results revealed that the sensitivity and dynamic range of the as-prepared sensor were 1.05 kPa^−1^ and 1 Pa to 2 kPa, respectively, which were comparable to the state-of-the-art ones; practical application measurements further indicated that the capacitive FPS was capable of detecting bending, finger tap, and human speech as well as identifying object profile; therefore, it shows good potential for applying in artificial skin and wearable devices.

## Introduction

Thanks to their flexibility and ease of integration into curved surfaces like human body, flexible pressure sensors (FPS) have received tremendous attentions and show great potentials for applications in wearable devices [1, 2], electronic skins [3, 4], biomedical systems [5], and human motion detection [6–9]; lots of structures and mechanisms such as field effect transistor [10, 11], capacitor [2, 12], piezoelectric effect [13–16], and piezoresistance effect [17–19] have been proposed to realize FPS; among them, the capacitive FPS becomes increasingly attractive due to its simple structure [20], large dynamic range [21], and good stability [22]. In terms of materials used in FPS, polydimethylsiloxane (PDMS) is an excellent material due to its good flexibility, biomedical compliance with human tissue, and dielectric property, and it is accordingly often used as a structural material in FPS as well as other flexible sensors [23–25]; in capacitive FPS, PDMS was often used as the dielectric layer [20, 26] and electrode substrate [2, 21]. When it comes to the electric conducting layer in FPS, silver nanowires (AgNWs), which have large potential and been widely used in flexible electronics such as solar cells [27–32] and film heaters [33, 34] due to their excellent electrical, optical, and mechanical properties, were often used together with PDMS; for example, Chen et al. [35] prepared silver nanowire (AgNWs)/(PDMS) composite films by partly embedding AgNWs in the PDMS layer to create rough surface, and the fabricated sensor device was able to achieve sensitivity three times of that using common metal film electrodes. Yao et al. [2] firstly achieved parallel AgNW lines on silicon through a pre-patterned PDMS shadow mask; then, they casted a liquid PDMS onto the AgNW-silicon substrate; by peeling off the PDMS after cured for 12 h, an AgNW-embedded PDMS electrode was obtained; finally, a capacitive FPS was fabricated which successfully detected thumb movement, knee joint strain, and other human motions.

To achieve high sensitivity of capacitive FPS, it is usually necessary to produce micropatterns in the dielectric layer and/or electrodes, and complicated procedures, like micromachining [2, 35, 36] as well as micromolding [7, 21, 26], are often involved; for example, Bao et al. [26] created inversed pyramid patterns in silicon and then transferred the patterns to PDMS by casting it on the silicon mold; the pyramid patterns were therefore produced on PDMS. Li et al. [21] also used molding technique to create an inverse structure of lotus surface on PDMS ensued by depositing a thin gold layer as the electrode, and a capacitive FPS were fabricated by using polystyrene (PS) microspheres as the dielectric layer sandwiched by two PDMS electrodes. In this work, a much simple procedure was proposed by using an ordinary printing paper deposited with AgNWs as the electrode substrate, and high-performance capacitive FPS was constructed by using PDMS as the dielectric layer laminated with AgNW-paper substrate on both sides; the test results demonstrated that the sensitivity and dynamic range of device were 1.05 kPa^−1^ and 1 Pa to 2 kPa; furthermore, it was capable of identifying object shape, finger tap, and voice-induced vibration, showing its good potential for artificial skin and wearable devices.

## Methods

### Preparation of AgNWs, AgNWs Films, PDMS Films, and Capacitive FPS

AgNWs were synthesized by hydrothermal method: first, 0.3 mole per liter (M) solution of polyvinyl pyrrolidone (PVP) (molecular weight 30000)/ethylene glycol (EG) was prepared by adding 0.2 g PVP into 6 ml EG, and the mixture was stirred for 20 min; similarly, 0.1 M solution of AgNO_3_/EG and 0.01 M sodium chloride (NaCl)/EG were also prepared. Second, solutions of AgNO_3_/EG and NaCl/EG were added into PVP/EG and stirred until uniform solution was obtained, which was then transferred to a Teflon lining and put into the reaction kettle; the kettle was heated to 140 °C for 2 h and then to 160 °C for 30 min for the growth of AgNWs; after the kettle was naturally cooled down to room temperature, AgNWs in the form of white powder were obtained by washing and centrifugal filtering the products sequentially with acetone and deionized water for three times. Lastly, the obtained AgNWs were ultrasonically dispersed in 100 ml ethanol for AgNW film preparation.

Preparation techniques including airbrush spraying, spin-coating, and soak-coating were used for AgNWs films, and experimental results reveal that the airbrush spraying one has the advantage of high efficiency, good uniformity, and adherence; the details of the AgNW film preparation were as follows: a clean printing paper with the size of 20 mm × 20 mm was used as the substrate which was put on a hotplate at 100 °C; the diameter of the out port of the airbrush was 0.5 mm, the distance between the airbrush and substrate was 150 mm; the preset pressure of airbrush was 0.1 MPa; AgNWs with different thickness and electric resistance can be obtained by adjusting the spraying time; after deposition, the substrate was kept on the hotplate for 1 h to completely remove PVP around AgNWs. PDMS were prepared from Sylgard 184 precursors (Dow Corning). Firstly, the main and curing agents of the precursors were mixed with a mass ratio of 10:1; after stirring for 20 min, the mixture was vacuumed for 10 min to remove bubbles during stirring; then, it was spin-coated on a carefully cleaned glass substrate. The substrate was then annealed at 65 °C for 2 h to form cured PDMS, and a freestanding PDMS film can be finally obtained by peeling it off the glass substrate.

**Fig. 1:**
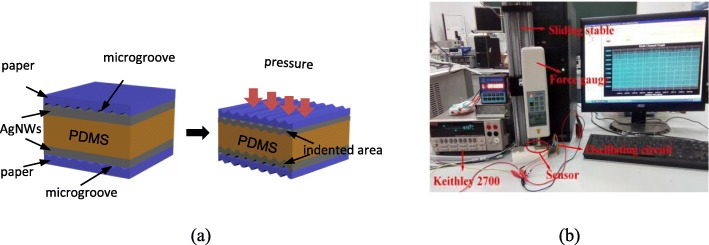
**a** Structure of AgNW-paper-based capacitive FPS and simplified mechanism. **b** Test platform for capacitive FPS

The sandwich-type capacitive FPS (Fig. 1) was fabricated by using two AgNW-paper substrates were used as the electrodes and PDMS as the dielectrics; electric signals were extracted out by two copper wires, which were glued on the electrodes by conductive silver paint; finally, the sensor was packaged by transparent tape.

### Characterization and Test

Surface morphologies of AgNWs and papers were characterized by scanning electron microscope (SEM) (type Inspect F50, FEI, US); Ultraviolet-visible (UV-Vis) spectroscopy tests (SHIMAZU 1700, Japan) were performed to analyze the composition of the AgNWs; for sensor test, a pressure stimulating platform was built based on a force gauge (HP-5, Yueqing Handpi Instruments Co., Ltd, China); a homemade oscillating circuit based on the LM555 timer model was designed to convert capacitance variation to frequency one; data acquisition was realized on a personal computer via Keithley2700 multi-meter (Keithley, USA).

## Results and Discussions

**Fig. 2:**
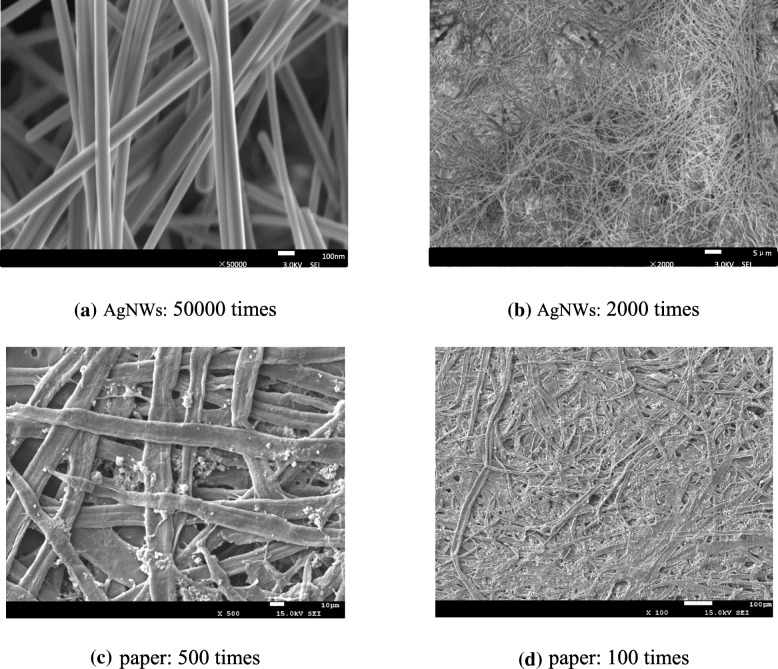
**a**–**d** SEM photos of AgNW film and paper

**Fig. 3:**
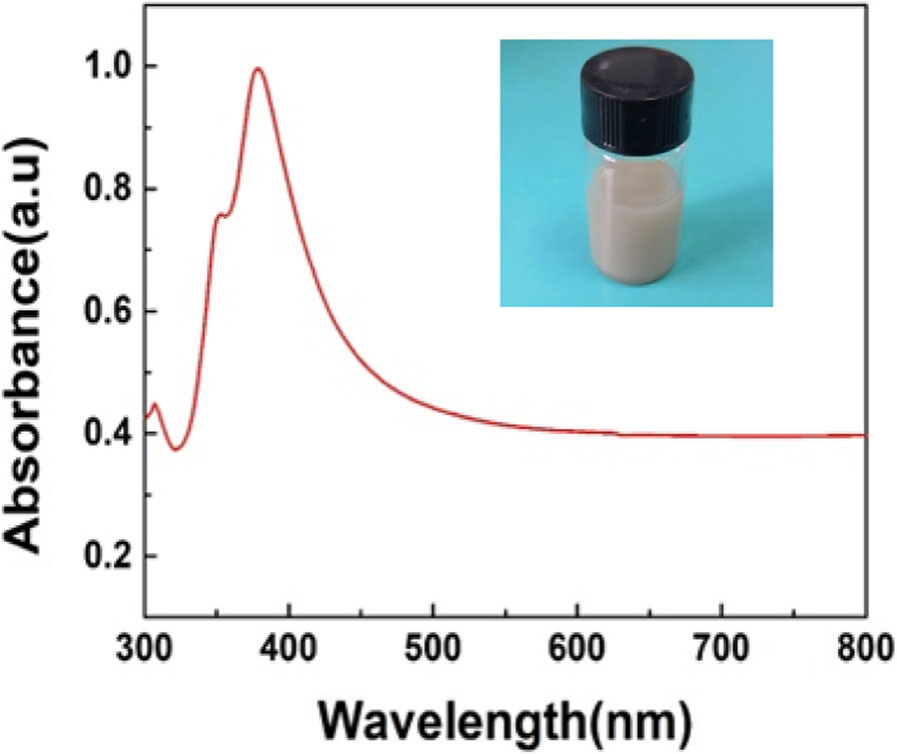
UV-Vis spectrum of the AgNWs

As shown in Fig. 2a, SEM photo indicates that the prepared AgNWs have uniformly long and thin shapes with diameters about 100 nm, and no impurities are found in the film; Fig. 2b implies the film has a relatively high density which will help to achieve highly conductive electrodes of the capacitive FPS. To further investigate the purity of AgNWs, UV-Vis spectrum was tested as given in Fig. 3. It clearly demonstrates that two peaks at 355 nm and 380 nm appear in the absorption curves, which are due to transverse and longitudinal plasmon resonances of AgNWs; other character peaks or noises would also appear if impurities like AgCl, AgNO_3_, or silver nanoparticles exist in the film; therefore, the UV-Vis spectrum further proves that high-quality AgNWs were successfully synthesized.

**Fig. 4:**
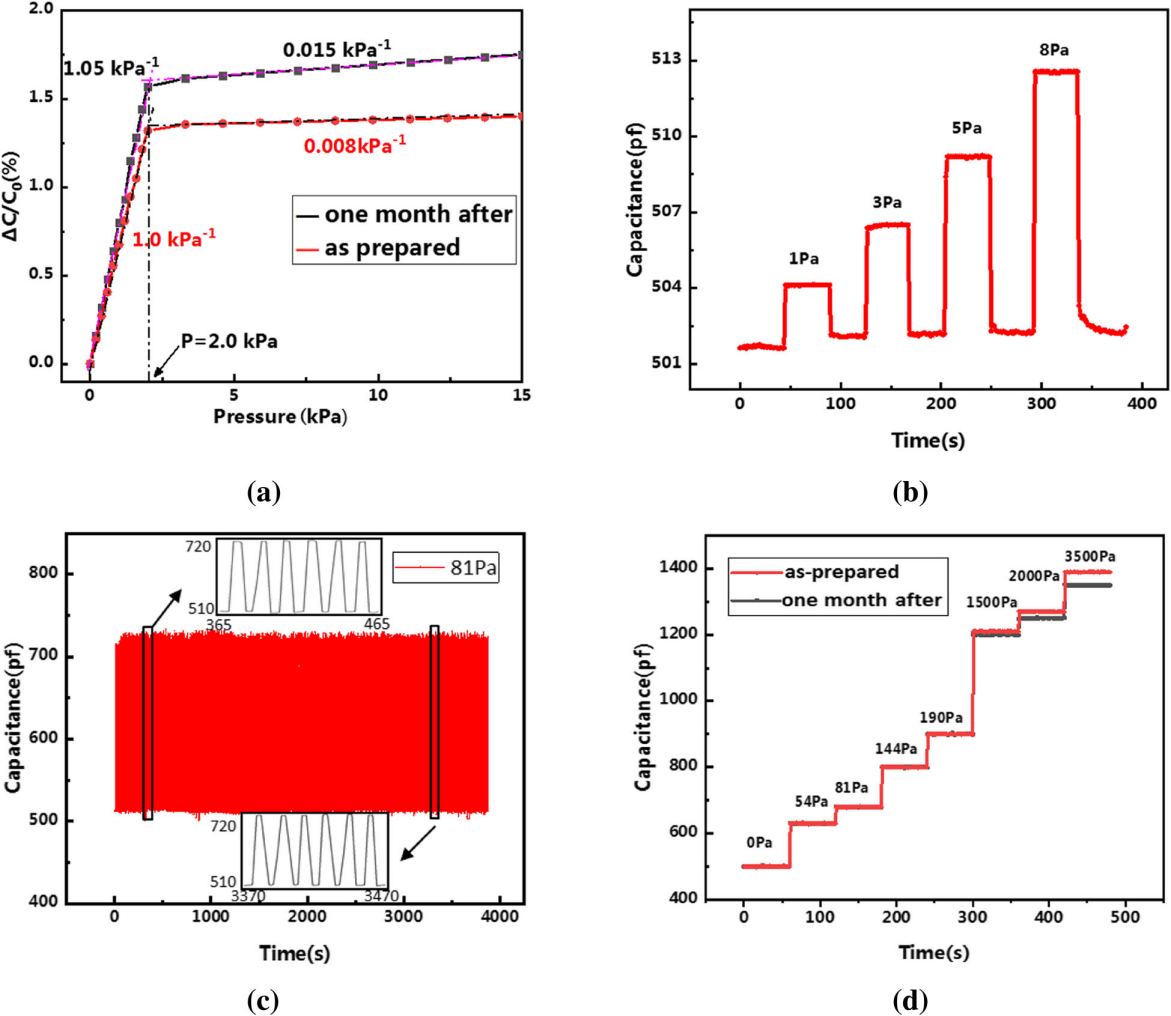
Response test of AgNW-paper-based capacitive FPS: **a** pressure-capacitance relations within a large pressure range, **b** response at low pressure, **c** repetitive test in short duration, and **d** aging performance after one month

Figure 4 gives the response test results of the sample; as shown in Fig. 4a, the response curve can be approximately divided into two linear parts in the whole pressure range, i.e., a highly sensitive part at low pressure and a lowly sensitive part at high pressure with the turning point located at 2 KPa. This phenomenon is common for capacitive FPS especially based on PDMS [21, 22] which can be interpreted as follows: the PDMS has its elastic limit; as the applied pressure is low, the PDMS shows good elasticity, large strain can be produced, and large capacitance variation (Δ*C*) can therefore be expected; while as applied pressure gets large enough, PDMS becomes so dense that it is not elastic enough anymore, no obvious strain can be produced anymore by the applied pressure, and thus, only low sensitivity of the sensor can be reached, and as a consequence, only the high sensitivity range is used for measurement. From the data in Fig. 4a, it can be calculated that the capacitive FPS sample has a sensitivity as high as 1.05 KPa^−1^, this value is better than those reported in most literatures [12, 26, 37–39] and comparable to the results of AgNWs/microstructured PDMS-based capacitive FPS in our previous work [22], whilst the fabrication procedure is much simpler. The mechanism behind this good performance may be attributed to the morphological nature of the paper; as displayed in Fig. 2c and d, SEM photos of the paper reveal that a large number of micro-grooves and voids exist on the paper, since it is hard to compress the air in the micro-grooves and voids; the air will then move downwards and create numerous indents in the AgNWs when outside pressure is applied; these indents will finally transfer to the PDMS film due to high flexibility of both the AgNWs and PDMS; and as a result, equivalent areas of the electrodes increase as well as the distance between them decreases, both of which are useful for achieving a larger capacitance variation. More specific characterization of sensor performance in extremely low-pressure range was conducted as presented in Fig. 4b. It can be clearly seen that the sample is able to respond to a pressure as low as 1 Pa, demonstrating its high sensitivity; also, it can fully recover to its initial value after unloading from each pressure, reflecting its good stability and fast response speed. Figure 4c gives the result of short-term repeatability test where the sensor underwent pressing (81 Pa) and releasing continuously for 500 periods. The enlarged response curves at the beginning and the end show very similar trends, further implying the sample’s good stability and repeatability. Furthermore, long-term repeatability test was performed after 1 month; as shown in Fig. 4a, the response of the sample at low-pressure range does not change after 1 month; on the other hand, although the high-pressure response underwent an obvious decrease, as mentioned above, this would not influence the sensor’s performance. Figure 4d compares the sample’s performance before and after 1 month at some specific pressure values; it further reveals that no degradation of the device can be found at low pressure; on the other hand, although the response at high pressure decreases, it shows no variation under constant pressure, indicating the sample’s still-good stability.

**Fig. 5:**
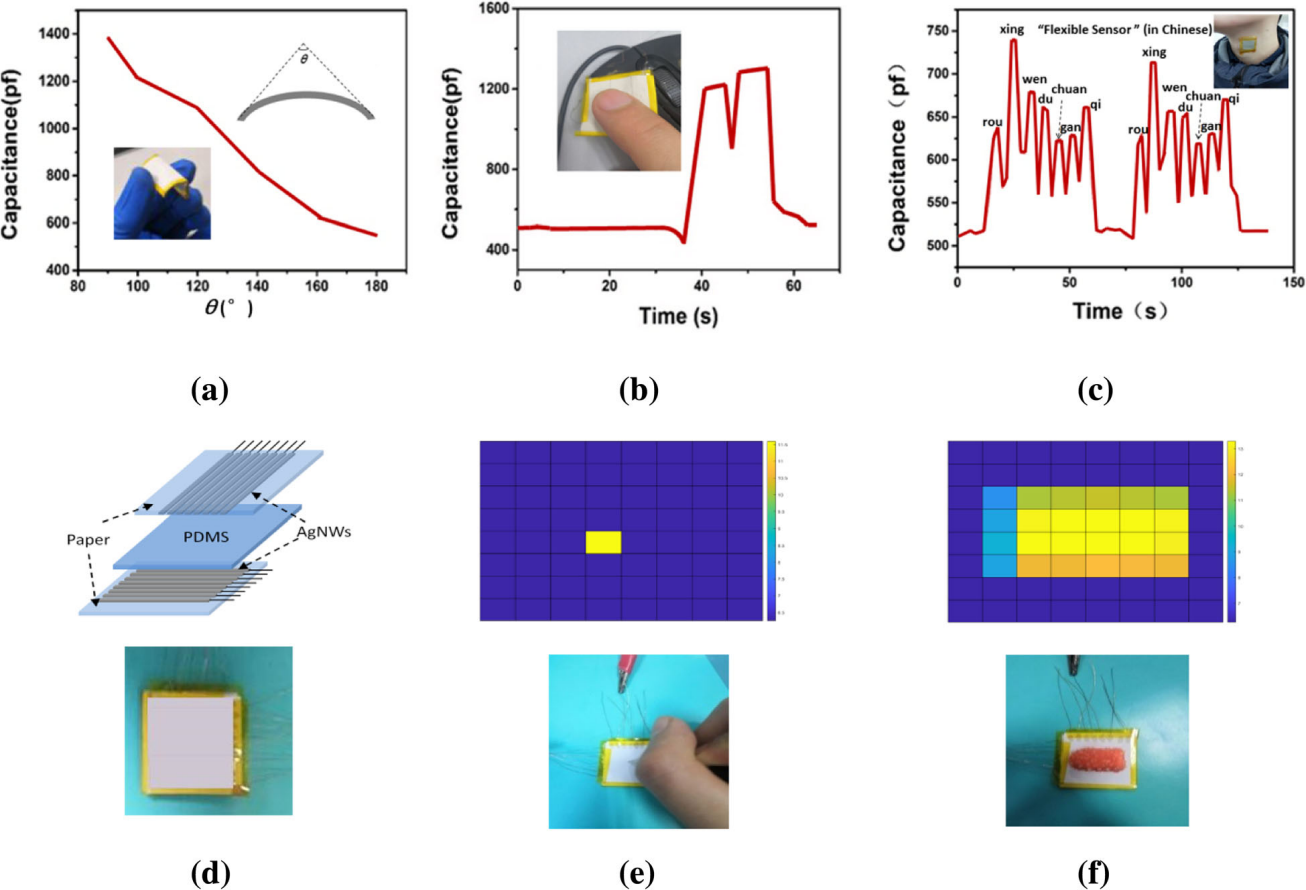
Applications of AgNW-paper-based capacitive FPS: **a** bending test, **b** finger tap test, **c** voice test, **d** 8 × 8 array of AgNW-paper-based capacitive FPS, **e** detection of a pencil tip, **f** detection of a bullet shape handmade from plasticine

To investigate the practicability of the AgNW-paper-based capacitive FPS, several real-life-related tests were performed. Figure 5a gives the result of the bending test; the bending angle theta, as shown in the inset, is defined as the included angle formed by the two lines tangent to the bending sensor at both ends. It reveals that the sample is very sensitive to the bending and the more bending the sample, the larger its capacitance; furthermore, the capacitance-theta curve interestingly has a nearly linear relation, which provides the sample a good potential for the measurement of bending status of the joints of the human body. Figure 5b shows the sensor can clearly detect double-click motion; the pressure during click can produce a capacitance variation as much as 700 pF, one time larger than its initial value; furthermore, as shown in Fig. 5c, the sensor can identify each syllable the experimenter says and demonstrates high sensitivity and excellent repeatability. To further explore the potential of the capacitive FPS, an 8 × 8 array of AgNW-paper capacitive FPS was fabricated as depicted in Fig. 5d; the electrode lines were formed by spraying the AgNWs through a hard mask and the size of the pixel was 2 mm × 2 mm. Figure 5e demonstrates that the array can easily detect a pencil tip, and since the tip was small enough, the neighboring pixels were not affected at all, showing its negligible crosstalk effect; additionally, as shown in Fig. 5, after a plasticine-made bullet was placed on the array, it was capable of recognizing the bullet’s shape; specifically, the mapping results imply that most mass of the bullet locates at the central two rows, whilst the second column of pixels on the left has the smallest signals due to small mass at the bullet head.

## Conclusion

By using an ordinary paper as the substrate, AgNWs were prepared via hydrothermal synthesis technique. SEM and UV-Vis characterization results indicated the AgNWs have uniform size, large length-to-diameter ratio, and high purity, which are desirable for good flexibility and electric conductivity of the AgNWs. Capacitive FPS sample was prepared by using the AgNW-paper substrates as electrodes and PDMS as dielectrics; performance tests demonstrated that the sample had not only high sensitivity and large dynamic measurement range, but also good stability and repeatability. In addition, the sample shows its capability in detecting human motions such as joint bending, finger tapping, and speech; furthermore, an 8 × 8 array of capacitive FPS with pixel size of 2 mm × 2 mm was fabricated, and the results showed that the array has high sensitivity, negligible crosstalk effect, and potential for object profile identification. These tests indicate our AgNW-paper capacitive FPS has good potential for applications such as artificial skin, movement monitoring, wearable device, and object identification.

## Data Availability

The datasets used and/or analyzed during the current study are available from the corresponding author on reasonable request.

## Abbreviations

FPS: Flexible pressure sensors
AgNWs: Silver nanowires
M: Mole per liter
PVP: Polyvinyl pyrrolidone
PS: Polystyrene
EG: Ethylene glycol
NaCl: Sodium chloride
SEM: Scanning electron microscope
UV-Vis: Ultraviolet-visible
Δ*C*: Capacitance variation
pF: Picofarad
